# Decreased Nursing Staffing Adversely Affects Emergency Department Throughput Metrics

**DOI:** 10.5811/westjem.2018.1.36327

**Published:** 2018-04-05

**Authors:** Zachariah Ramsey, Joseph S. Palter, John Hardwick, Jordan Moskoff, Errick L. Christian, John Bailitz

**Affiliations:** * John H. Stroger Hospital of Cook County, Department of Emergency Medicine, Chicago, Illinois; † Rush Medical College, Department of Emergency Medicine, Chicago, Illinois; ‡ Northwestern Memorial Hospital, Department of Emergency Medicine, Chicago, Illinois

## Abstract

**Introduction:**

The effect of nurse staffing on emergency department (ED) efficiency remains a significant area of interest to administrators, physicians, and nurses. We believe that decreased nursing staffing adversely affects key ED throughput metrics.

**Methods:**

We conducted a retrospective observational review of our electronic medical record database from 1/1/2015 to 12/31/2015 at a high-volume, urban public hospital. We report nursing hours, door-to-discharge length of stay (LOS) and door-to-admit LOS, and percentage of patients who left without being seen (LWBS). ED nursing hours per day was examined across quartiles with the effect evaluated using analysis of covariance and controlled for total daily ED volume, hospital occupancy and ED admission rate.

**Results:**

From 1/1/15–12/31/15, 105,887 patients presented to the ED with a range of 336 to 580 nursing hours per day with a median of 464.7. Independent of daily ED volume, hospital occupancy and ED admission rate, days in the lowest quartile of nursing hours experienced a 28.2-minute increase per patient in door-to-discharge LOS compared to days in the highest quartile of nursing hours. Door-to-admit LOS showed no significant change across quartiles. There was also an increase of nine patients per day who left without being seen by a provider in the lowest quartile of nursing hours compared to the highest quartile.

**Conclusion:**

Lower nursing hours contribute to a statistically significant increase in door-to-discharge LOS and number of LWBS patients, independent of daily ED volume, hospital occupancy and ED admission rate. Consideration of the impact of nursing staffing is needed to optimize throughput metrics for our urban, safety-net hospital.

## INTRODUCTION

Emergency department (ED) efficiency remains a vital aspect of delivering safe, quality care. ED utilization has risen considerably without a corresponding rise in available emergency services.^1^,^2^ To respond to the increased demand, it is imperative to identify factors that contribute to delays in care. Researchers have identified several hospital characteristics associated with worse ED throughput or ED time on ambulance diversion including ED crowding,^3^ percentage of ED patients admitted,^4^,^5^,^6^ number of elective surgical admissions,^5^ hospital occupancy,^5^,^6^,^7^ training level of the treating physician,^3^ access to expedited diagnostic testing,^8^ socioeconomic status of the surrounding neighborhood,^9^ and decreased nurse staffing.^10^

Prior studies identified that increased nurse-to-patient ratios correlate with improved patient outcomes^11^,^12^ and that lower staffing is associated with increased left without being seen (LWBS) rates^13^ and increased ED care times.^10^ Our urban, tertiary care, safety-net, teaching hospital suffered a nursing shortage during 2015 due to an administrative initiative to decrease costs by limiting nurse overtime hours. Without a concomitant increase in hiring, this change caused significant gaps in ED nurse staffing. These gaps led to unpredictable closures of sections of the ED and increased average nurse-to-patient ratios. Our goal was to evaluate the impact of decreased nurse staffing on ED throughput metrics. We believe decreased nurse staffing adversely affects these metrics.

## METHODS

Our hospital is an urban, tertiary care, safety-net hospital with 254 medical/surgical inpatient beds and 80 ED beds. The ED is staffed by full-time, board-certified attending emergency physicians who supervise emergency medicine residents, residents from other specialties, and physician assistants. Hospital-stipulated maximum nurse-to-patient ratios were not changed or exceeded during the study period. Nurses work a mix of 8- and 12-hour shifts. The ED is also staffed by patient care technicians and patient transporters.

We conducted a retrospective observational review using Cerner First Net electronic medical record (EMR) database. All EMRs of 105,887 ED visits from January 1, 2015, to December 31, 2015, were queried after institutional review board approval. We included in the analysis all patients discharged or admitted to the medical/surgical inpatient beds in the analysis regardless of inpatient or observational status. Patients admitted to the intensive care unit or the ED observation unit were excluded as the admission protocol to these units varies significantly from general admission; therefore, we could not accurately capture the length of stay (LOS) of these patients from EMR review. A total of 6,602 patients were excluded.

The unit of measure was a 24-hour period starting at midnight. Daily number of patients admitted, discharged, and LWBS as well as the total daily volume in the ED was recorded. Daily nursing hours were determined from nursing staff records for each shift and summed for each day. We measured door-to-discharge LOS in minutes as the interval from the time of presentation to the ED to when the provider discharged the patient. We captured the time of initial presentation by the time the patient was registered at the front desk. The time of discharge was captured by a physician order for discharge placed in the EMR. Door-to-admit LOS was measured in minutes as the interval from the time of ED presentation to when the nurse placed an electronic order that the patient was ready to be transported to the ward. We defined hospital occupancy as the sum of the number of patients in a hospital bed at midnight and the number of patients discharged in the preceding 24 hours divided by the total number of hospital beds. This method was used previously by Forster,^7^ which helps capture the true use of inpatient beds during a 24-hour period.

We evaluated the effect of ED nursing hours on throughput metrics using analysis of covariance and controlled for total daily ED volume, hospital occupancy and admission rate. Daily nursing hours were compared across quartiles as a fixed factor. We used daily door-to-discharge LOS, door-to-admit LOS, and the number of patients who LWBS as the dependent variables in each model. SPSS Univariate GLM procedure was used for all analyses.

## RESULTS

The mean number of visits per day was 290 with a range of 129 – 425. Nursing hours ranged from 336 – 580 nursing hours per day with a median of 464.7. The daily mean LOS for discharged patients was 249.8 minutes, and the range was 155 – 389. The daily mean LOS for admitted patients was 441.5 minutes, and the range was 259 – 796. The ED mean admission rate was 17.5% with a range of 10.8% – 23.9%. The daily mean of patients that LWBS was 17.5 and totaled 6,387 with a range of 1 – 55 patients per day. The daily mean hospital occupancy was 98.3%, and the range was 68.5% – 116.3%. The figure depicts the daily mean LOS for discharged and admitted patients as well as nursing hours by date throughout the course of the study.

Outcome variables are summarized in the table. ED door-to-discharge LOS and the number of patients who LWBS were both significantly affected by a decrease in daily nursing hours independent of ED daily volume, hospital occupancy and admission rate. Days in the lowest quartile of nursing hours experienced a 28.2-minute increase per patient in door-to-discharge LOS compared to days in the highest quartile of nursing hours. Across these same quartiles, days in the lowest quartile of nursing hours observed an increase of nine patients that LWBS per day. Both these differences were statistically significant. Door-to-admit LOS was not significantly affected by nursing hours. In the case of door-to-discharge LOS and number of patients that LWBS, while comparing adjacent quartiles did not always lead to statistically significant differences, there was a clear trend in the data across the quartiles that showed correlation.

## DISCUSSION

Often, ED throughput metrics are equated to ED performance metrics. Thus, we are constantly seeking to understand the factors that impact our facility’s performance. One of those factors in our study was nurse staffing. Suboptimal nurse staffing may impact a number of nursing tasks such as triage, vital signs, phlebotomy, medication administration, procedures, and discharge education. As nursing delays accumulate, this translates into longer wait times, leading to more patients who LWBS. It is likely that nurse staffing levels affect all important steps in a patient’s path through the department^13^ and has previously been shown to impact patient safety.^11^,^12^ Cost analysis may delineate whether increased nurse staffing drives up front-end costs, but also generates additional revenue through more patient evaluations and decreased LWBS rates. Ultimately, expenses and revenue related to staffing and throughput are likely institutionally specific, but it is an important consideration nonetheless.

Nationwide nursing shortages continue to be an ongoing issue. High nursing turnover, changes to overtime rules and lengthy hiring processes, among other factors, can all contribute to nursing shortages and decreased nursing hours in EDs. Our study further contributes to the body of evidence that decreased nurse staffing directly contributes to the number of patients who LWBS and increased ED LOS, which is also shown to decrease patient satisfaction.^15^ Chang showed that organizational characteristics associated with decreased ED LOS included executive leadership involvement, hospital-wide coordinated strategies, data-driven management, and performance accountability.^16^ Our study provides additional data that may help providers further engage hospital administration to supply adequate nurse staffing that allows EDs to better achieve performance goals and improve the patient experience.

## LIMITATIONS

The authors were not blinded to the hypothesis of this retrospective study during data abstraction; therefore, selection of controls was subject to author bias. The computer-derived data allowed for large data collection, but also contributed to our limitations. It is unknown when discharged patients received final instructions from nurses and hence physically left the ED, as this is not captured in our EMR. We did not address acuity of illness or triage scoring directly, an independent determinant of ED throughput metrics,^14^ but rather used the surrogate of ED admission rate. Our analysis only measured data over 24-hour periods. It is possible that certain shifts were affected disproportionately by the decreased nurse staffing.

As a single institution study in an urban, tertiary care, safety-net hospital, our results may not be generalizable to other settings, specifically smaller-volume EDs with smaller nursing staffs. Our hospital spends negligible time on diversion each year so this was not included as a factor, though previous studies^4^ ^5^ ^14^ revealed diversion correlates with worsened throughput performance. Lastly, because the statistical method was designed to show correlation rather than causation, other confounding factors may contribute.

## CONCLUSION

Decreased nursing hours correlated to an increased ED LOS for discharged patients and increased LWBS rate. This analysis is a pivotal step in identifying and ensuring appropriate nurse staffing to optimize ED quality metrics. Further analysis may illustrate an ideal number of nursing hours per day for maximum benefit, but would likely require breaking down data into specific shifts. Future research may examine the cost impact of increased nursing hours compared to lost revenue from patients who LWBS. Finally, understanding the impact of nurse staffing on patient satisfaction is another area ripe for further study.

## Figures and Tables

**Figure:**
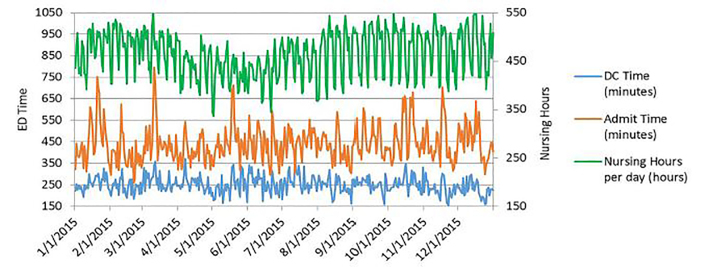
Daily mean length of stay for discharged and admitted patients as well as daily nursing hours by date. *ED*, emergency department; *DC*, discharge.

**Table:** Outcome variables.

	Mean ED LOS for discharged patients (n=74,951)	Mean ED LOS for admitted patients (n=18,487)	Mean LWBS per day (n=6,387)
1st Quartile nursing hours (336 – 422)	265.0 Minutes (95% CI [256.4 – 273.6])	454.7 Minutes (95% CI [436.6 – 472.7])	22 Patients (95% CI [20 – 24])
2nd Quartile nursing hours (423 – 472)	257.4 Minutes (95% CI [250.6 – 264.2])	445.6 Minutes (95% CI [431.4 – 459.7])	20 Patients (95% CI [19 – 21])
3rd Quartile nursing hours (473–504)	238.9 Minutes (95% CI [231.6 – 246.2])	429.2 Minutes (95% CI [414.0 – 444.4])	15 Patients (95% CI [13 – 16])
4th Quartile nursing hours (505–580)	236.8 Minutes (95% CI [229.0 – 244.5])	436.1 Minutes (95% CI [420.0 – 452.4])	13 Patients (95% CI [12 – 15])

Covariates appearing in the model are evaluated at the following values: Daily ED Volume = 290.3, Hospital Occupancy = 249.7 (98.3%),

ED Admission Rate = 17.5%.

*ED*, emergency department; *LOS*, length of stay; *LWBS*, left without being seen; *CI*, confidence interval.
